# Impact of Pharmacists’ Interventions and Patients’ Decision on Health Outcomes in Terms of Medication Adherence and Quality Use of Medicines among Patients Attending Community Pharmacies: A Systematic Review

**DOI:** 10.3390/ijerph18094392

**Published:** 2021-04-21

**Authors:** Kingston Rajiah, Shreeta Sivarasa, Mari Kannan Maharajan

**Affiliations:** 1 Department of Pharmacy Practice, School of Pharmacy, International Medical University, Kuala Lumpur 57200, Malaysia; kingrajiah@gmail.com; 2 Student, Master in Pharmacy Practice, School of Postgraduate Studies, International Medical University, Kuala Lumpur 57200, Malaysia; SHREETASIVARASA@student.imu.edu.my

**Keywords:** pharmacist interventions, patient choice, patient decision, quality use of medicine, patient-centered care, patient autonomy

## Abstract

Community pharmacists are responsible for providing the appropriate information on the use of medications to patients, which may enhance their medication adherence. The extent of control that patients have on their health care preferences creates many challenges for community pharmacists. This study aimed to determine the impact of pharmacist interventions and patient decisions on health outcomes concerning medication adherence and the quality use of medicines among patients attending community pharmacies. Appropriate studies were identified in a systematic search using the databases of Medline, Scopus, Google Scholar, and PubMed. The search included literature published between 2004 and 2019. The database searches yielded 683 titles, of which 19 studies were included after the full-text analysis with a total of 9313 participants. Metaprop command in Stata software version 14 was used for the analysis. This study was undertaken based on the general principles of the Cochrane Handbook for Systematic Reviews of Interventions and subsequently reported according to the Preferred Reporting Items for Systematic Reviews (PRISMA) extension. The Grading of Recommendations, Assessment, Development and Evaluations (GRADE) approach was directly used to rate the quality of evidence (high, moderate, low, or very low). The results revealed the effective interaction between patients and community pharmacists, the importance of pharmacist intervention on medication adherence and quality use of medicine, and the role of community pharmacists in counselling patients. Decision/choice of patients in self-care and self-medication is a factor contributing to health outcomes. Effective interaction of community pharmacists with patients in terms of medication adherence and quality use of medicines provided a better health outcome among patients. The community pharmacists influenced the decision/choice of patients in self-care and self-medications.

## 1. Introduction

The pharmacy profession plays an important role in the health care setting and pharmacists undertake a range of responsibilities in health care services [1]. The services provided in community pharmacy have undergone rapid expansion in recent years; these include public health advice on disease management and prevention, counselling on the rational use of medicines, and making appropriate referrals to other relevant health care professionals [2]. Community pharmacists are often the first point of contact for reliable information and advice for consumers and patients [3].

Patient-centered communication in community pharmacy facilitates patient empowerment and encourages patients to take an active part in the decision-making concerning their care. Patient empowerment is an essential prerequisite for effective autonomy [4] and involves the amount of control that patients have over their health outcomes [5]. On the other hand, autonomy, a crucial ethical and legal principle, allows individuals to make their own choices about their health behavior and the management of their illnesses [6]. This encompasses the individuals’ self-governance, the functional capacity to think, and to diligently decide and act based on the influence of independent thought [7]. In health care, patient autonomy refers to the patient’s personal right to consent or refuse a treatment [4].

Physical and mental competencies are necessary for patient empowerment, which creates dilemmas for health care providers in determining patient competencies [8]; in some instances, the patients’ personal ability to consent is questioned due to their lack of competencies [9]. As community pharmacists deal with patients while making health care decisions, the goal is to move toward optimal patient care [10]. In general, community pharmacists constantly face dilemmas in balancing the patients’ legal rights to autonomy against making interventions toward quality health outcomes [11]. However, in a health care intervention, the patient should have the capacity, be free from coercion or undue influence, and be informed appropriately about treatment goals [12]. This raises the question of whether the quality of patient health outcomes is due to the intervention of community pharmacists or the patients’ choices/decisions.

Pharmacists exert autonomy by placing patients’ rights before the regulations that govern pharmacy practice [13]. The amount of control that patients should have over their health care decisions/choices [14] is a cardinal question, which brings challenges to the community pharmacists.

In general, community pharmacists perceive that patients’ decision-making about their health care depends on their ideas and emotional state of social well-being [15]. This may put patients at higher risk if they are unaware of the medication dose regimen and the potential interactions with other medications [16]. Subsequently, this may lead to poor adherence to therapy and medication-related risks. A systematic review has already shown that community pharmacists play a major role in the health outcomes of patients by their interventions in medication adherence and quality use of medicines [17]. However, patient autonomy in health outcomes has not been analyzed among patients attending a community pharmacy. Therefore, there is a need for systematic information to measure the impact of pharmacists’ intervention and patients’ decisions on medication adherence and the quality use of medicines. Thus, the main goal of the present study was to determine the effect of patient autonomy on the health outcome of patients attending community pharmacies. This systematic review also aimed to summarize the evidence of pharmacists’ interventions and patients’ decisions on the health outcomes in terms of medication adherence and quality use of medicines among patients attending community pharmacies.

## 2. Materials and Methods

### 2.1. Ethics Approval

This study obtained ethical approval from the International Medical University (IMU) Joint Committee on Research and Ethics. Approval number: MPP1/202 (01).

### 2.2. Study Design

The impact of patient autonomy on medication adherence and quality use of medicine among patients attending community pharmacies was undertaken based on the general principles of the Cochrane Handbook for Systematic Reviews of Interventions and subsequently reported according to the Preferred Reporting Items for Systematic Reviews and Meta-Analyses (PRISMA) extension. The Grading of Recommendations, Assessment, Development and Evaluation (GRADE) approach was directly used to rate the quality of evidence (high, moderate, low, or very low) to determine if the authors provided a clearly defined research question and theoretical background.

### 2.3. Inclusion Criteria

Only peer-reviewed articles written in English and dealing with adult patients were considered. The studies were required to have an observational or experimental design and to address the potential relationship between the health outcomes and the patients’ decisions, with patients’ decisions being considered as an independent variable or as a mediator, and with health quality outcome being assessed directly as an outcome variable. The review was limited to the following in relation to medication adherence and quality use of medicine among patients attending community pharmacies:

- Effect of patients’ decision on health outcome.
- Effect of pharmacists’ intervention on health outcome.
- Effect of patients’ decision over pharmacists’ intervention on health outcome.

### 2.4. Exclusion Criteria

The studies excluded from this review were papers that were not available in English, qualitative studies, essays, literature reviews, commentaries, study protocols, conceptual papers, and conference abstracts.

### 2.5. Search Strategy

Given the importance of including evidence from observational studies in systematic reviews, relevant studies were identified through a systematic search of Medline and Scopus databases using subject headings and free-text terms. The PICO and Boolean search strategies were applied. The database search fields for titles, index terms, and abstracts were searched based on the following general research string: patient self-efficacy OR patient choice OR patient preference OR decision-making AND medication adherence AND quality use of medicines AND patient-centered care AND community pharmacist* AND pharmacist intervention. The search included all the literature published from 2004 to 2019. Subsequently, to supplement the main results of the online database searches (Medline and Scopus), the authors searched for articles that cited the potential identified studies using Google Scholar and PubMed MeSH and screened these articles for potential studies. Additionally, the authors directly screened the bibliographies of the potential identified studies. Two authors screened the titles and abstracts. Two authors selected and included full-text articles; these two authors read all the published full texts of the potential papers to confirm the inclusion criteria. All disagreements were resolved by consensus. In line with this, the data extraction process was performed by an author using a basic data-extraction instrument that encompassed the author, the year of the study, the setting, involvement of participants, possible interventions assessed, direct outcome measures, and all the main and essential findings of the studies.

### 2.6. Data Extraction and Synthesis

**Figure 1:**
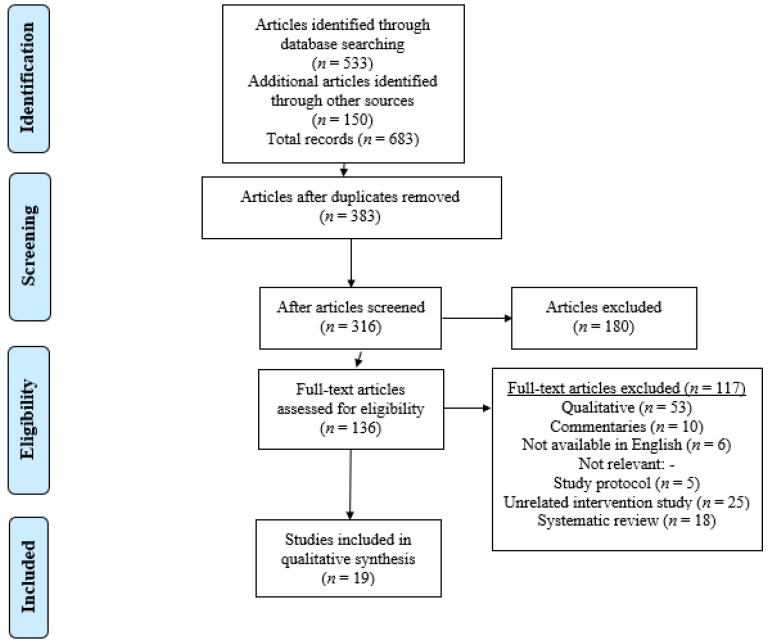
Flow diagram of the study selection process.

This systematic review was prepared following the PRISMA flow diagram (Figure 1) to assess the methodological quality of studies and sensitivity analysis of the GRADE approach. Cochrane’s Q and I2 statistics were used to evaluate the heterogeneity of the studies. Metaprop command in Stata software was used for the analysis (StataCorp. 2015, Stata Statistical Software: Release 14. College Station, TX, USA: StataCorp LP). Funnel plots by Egger and Begg tests were used to assess the publication bias. Forest plots showed high heterogeneity values, which resulted from variations in sample sizes and different populations. Hence, a meta-analysis was not used in the data synthesis as all the included studies directly demonstrated large heterogeneity in terms of the conceptualization and operationalization of quality health outcome in terms of medication adherence and quality use of medicine. Therefore, a descriptive synthesis was conducted and reported the impact of pharmacists’ interventions and patients’ decision on the health outcome. In this systematic review, effect sizes estimated the statistically significance studies, which were pooled and presented to obtain an overall effect size. The following four statements, which were common among published studies, were considered as outcomes for the systemic review:

1. Effective interaction between patients and community pharmacists.
2. Importance of pharmacists’ intervention on medication adherence and quality use of medicine.
3. Role of community pharmacists in counselling patients when dispensing medications.
4. Decision/choice of patients in self-care and self-medication.

The first statement assessed the effect of patients’ decisions on health outcomes, while statements 2 and 3 assessed the effect of pharmacists’ interventions on health outcomes. The last statement assessed the effect of patients’ decisions over pharmacists’ interventions on health outcomes.

### 2.7. Risk of Bias

The risk of bias in the studies was assessed, based on nine standard criteria suggested by the Cochrane Effective Practice and Organization of Care (EPOC) [18]. An overall rating of risk of bias was determined based on scores obtained for each domain. The risk of bias assessment is presented in Table 1.

## 3. Results

The database searches yielded 683 titles, of which 19 studies were included after the full-text analysis. If any of the articles reported more than one direct outcome regarding the relationship between the patient’s decision and quality health outcome, each result was included separately. Figure 1 provides an overview of the selection process.

### 3.1. Study Characteristics

The studies included in this systematic review (*n* = 19) were from various countries: the United Kingdom (*n* = 4), the United States of America (*n* = 3), Australia (*n* = 2), Saudi Arabia (*n* = 2), and Malta (2). There was one study each from Hungary, Canada, Bosnia-Herzegovina, Thailand, North Cyprus, and Kuwait. The characteristics of the included studies are described in Table 2.

### 3.2. Effective Interaction between Patients and the Community Pharmacists

**Figure 2:**
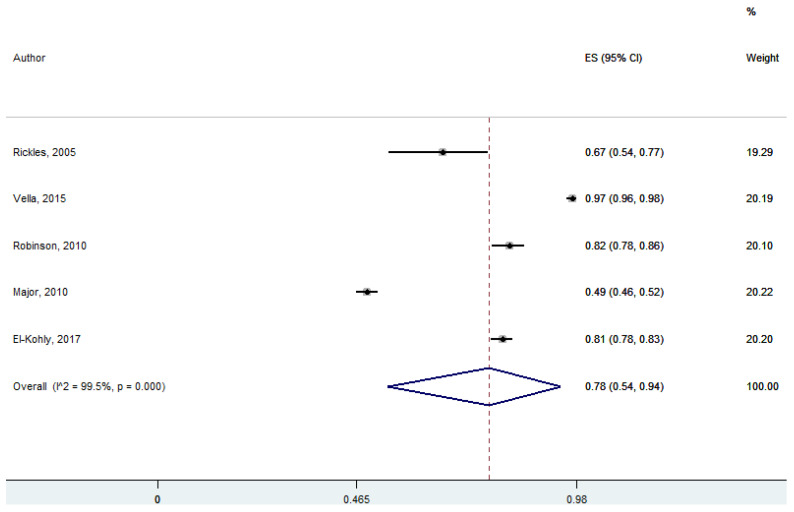
Effective interaction between patients and the community pharmacists (five studies).

Five studies discussed the effect of patient–pharmacist interaction. Pharmacists respected the covenantal professional relationship with their patients and had an effective interaction with them to achieve the desired health outcome by shared decision-making. Pharmacists had an effective interaction with their patients, which was statistically significant at 95% CI 47–85; *p* = 0.00 (pooled effect size: 0.67) (Figure 2).

### 3.3. Importance of Pharmacists’ Intervention on Medication Adherence and Quality Use of Medicine

**Figure 3:**
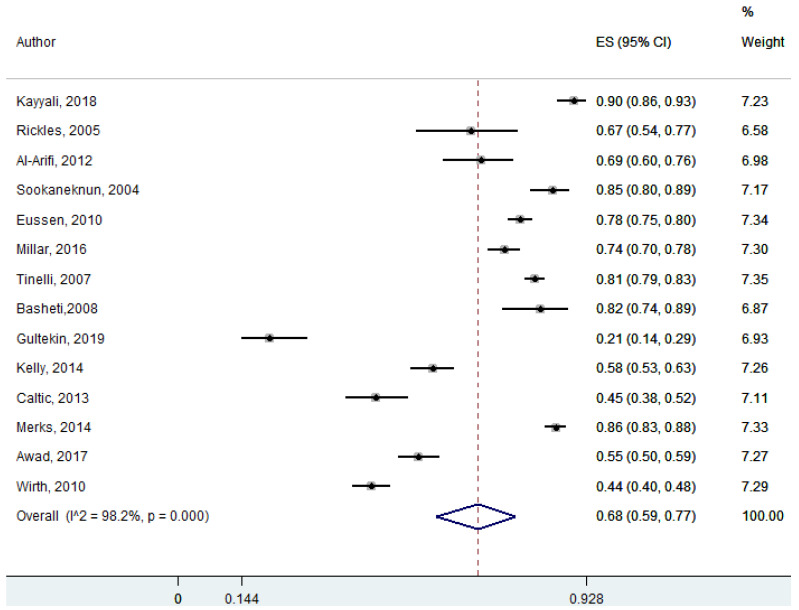
Importance of pharmacists’ intervention on medication adherence and quality use of medicine (14 studies).

Eight studies looked at the importance of pharmacists’ intervention on medication adherence and quality use of medicine. These studies revealed that patients considered pharmacists as important health care providers as pharmacists’ interventions provided a better health outcome and helped in decision making in terms of medication use and adherence. Patients agreed on the importance of pharmacists’ intervention on medication use and adherence, which was statistically significant at 95% Cl 39–66; *p* = 0.00 (pooled effect size: 0.53) (Figure 3).

### 3.4. Role of Community Pharmacists in Counselling Patients When Dispensing Medications

**Figure 4:**
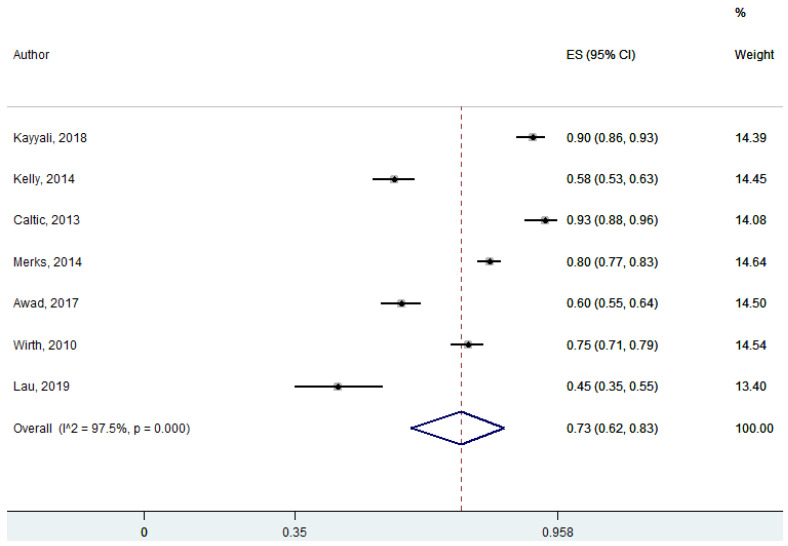
Role of community pharmacists in counselling patients when dispensing medications (seven studies).

Fourteen studies discussed the role of community pharmacists in dispensing prescription and non-prescription medications. The pharmacists had counselled the patients on medication adherence and quality use of medicines while dispensing medications for various ailments. Community pharmacists were involved in patient counselling in terms of medication adherence and quality use of medicines, which was statistically significant at 95% Cl 56–78; *p* = 0.00 (pooled effect size: 0.68) (Figure 4).

### 3.5. Decision/Choice of Patients in Self-Care and Self-Medication

**Figure 5:**
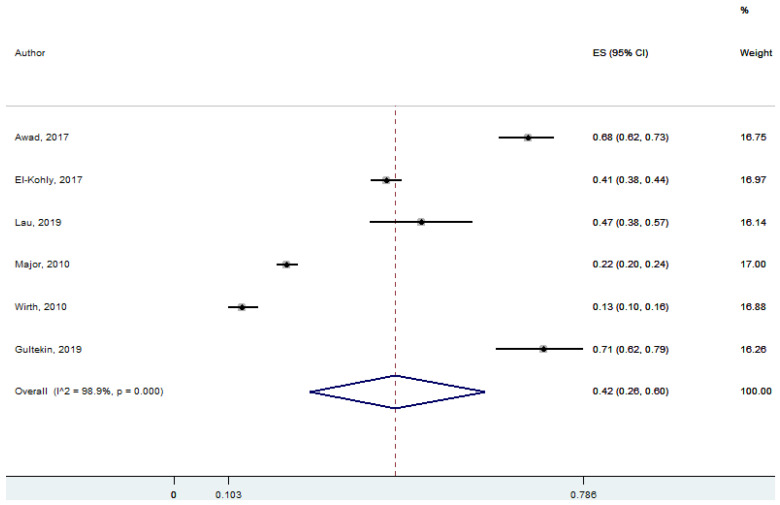
Decision/choice of patients in self-care and self-medication (six studies).

Six studies discussed the decisions/choices of patients in self-care and self-medication. Patients’ self-care directly related to their own decisions and the initiation of their actions by themselves. Patients’ preferences or choices sometimes depend on pharmacists’ recommendations while deciding on their medications for minor ailments. A vast number of minor ailments are self-limiting, requiring minimal or no medical interventions. Community pharmacists influenced the patients’ decision/choice in self-care and self-management, which was statistically significant at 95% Cl 26–60; *p* = 0.00 (pooled effect size: 0.42) (Figure 5).

## 4. Discussion

This systematic review was conducted to focus on health outcomes in terms of medication adherence and quality use of medicines in patients attending community pharmacies. The current evidence revealed that ‘Effective interaction between patients and the community pharmacists’, ‘Importance of pharmacists’ intervention on medication adherence and quality use of medicine’, ‘Role of community pharmacists in counselling patients when dispensing medications’, and ‘Decision/choice of patients in self-care and self-medication’ are factors that contributed to better health outcomes. Despite having different methodologies, most studies were consistent with the conclusion that the pharmacists’ interventions had an impact on patients’ decisions on the health outcome.

The results of this systematic review indicated an interesting aspect of ‘Patient–pharmacist interaction’. Patient–pharmacist interaction is about treating patients receiving health care with dignity and respect and involving them in all decisions about their health. It is an essential component in providing sufficient advice to patients on their medications. By establishing a rapport with patients, pharmacists provide advice on the rational use of medications [38]. This patient-centered approach and therapeutic relationship with patients has become the top priority for most health care professionals [39]. Building a good therapeutic relationship helps health care professionals to provide optimal care and quality health outcomes for patients [40]. The patient–pharmacist relationship enables patients to ask for advice and to share their decisions with their pharmacists and follow-up [41]. As the number of new medications available is increasing, and patients with long-term illness and comorbidity need to take more than one medication concurrently, it is challenging patients to comply with prescribed medication regimens [42]. Thus, pharmaceutical care is necessary for the quality use of medicine and adherence. Quality Use of Medicines is primarily a patient-oriented movement, with the documentation stressing the importance of patient–professional communication along with patient knowledge and understanding of their medicines. Studies have shown improved health outcomes when there is an effective interaction between pharmacists and patients [43,44]. Patients view their pharmacists’ competence as a key factor to sustain a therapeutic relationship with them. However, to have effective communication between patient and pharmacists, trust and patient satisfaction are necessary [45].

This systematic review highlighted the importance of pharmacists’ intervention on medication adherence and quality use of medicine in managing patients’ medication therapy. Medication therapy management helps pharmacists to identify patients at-risk and resolve their pharmaceutical care issues [46]. Community pharmacists educate their patients on the quality use of medicines by encouraging their patients to take correct dosages at the correct time; they advise patients on appropriate medication usage and other ways to manage their ailments [47]. There is a high prevalence of inappropriate prescribing and medication errors exist within health care systems [48]. This often leads to adverse events, which are usually preventable. Pharmacists have a significant impact on reducing and preventing medication errors and their related problems [49]. As pharmacists’ services and interventions are effective and contribute to the patients’ cost savings, they help to reduce health care expenses [50]. Pharmacists’ interventions increase patient adherence to medications, thereby reducing unnecessary hospital visits and hospitalizations [51].

The results from this systematic review also revealed that patient counselling by community pharmacists on medication adherence and quality use of medicines while dispensing medication influences the quality health outcome of the patients. The quality health outcome is associated with patient adherence to the treatment regimens. Moreover, non-adherence to treatment regimens may become a threat to health and an increased economic burden for the patients [52]. Using a patient-centered approach and effective communication, community pharmacists can help patients manage their condition appropriately, improve their health, and help them to minimize the disease burden. Nevertheless, community pharmacists also need to consider that any social and/or economic changes in a patient’s life may affect their medical management [53]. Hence, each encounter with patients must include an intervention comprising a review of medication and compliance problems; this may enhance the autonomy of patients in deciding/choosing their management therapy.

Despite the meaningful intention of promoting choice to patients, it is not always possible to do this, especially if we look at the implications of such choices from different perspectives. It is suggested that there is always a probable conflict between allowing a greater choice for consumers and changing health care policy that is directed more toward standardized health care provision. Pharmacists are expected to offer choice, simply because it will promote and enhance autonomy, but also it is the right thing to do. However, the choice itself comes with responsibility, that is, one that is accountable for their choice and decision-making, and arguably, one choice usually impacts on other people, particularly when resources are scarce. In the present review, patients’ decisions/choices in self-care and self-medication were found to affect their health outcome, especially for minor ailments. The availability of “over-the-counter” or “non-prescription” medications to the public may vary in different countries. Although these medications are approved by the regulatory agencies, their safety and effectiveness for individual consumers/patients often require supervision or intervention by health care professionals. While studies suggested that most consumers/patients read the information provided before taking medications [54], their decisions in selecting such medication may not be appropriate. Most non-prescription medications are purchased based on product information available on the packaging [22]. When consumers/patients seek help when purchasing, this is termed “facilitated self-medication” [55]. When medications are purchased through community pharmacies, pharmacists facilitate self-care decision making. As the use of over the counter or non-prescription medications is increasing, there is a need for specific counselling by pharmacists while facilitating self-medication [56].

## 5. Strengths and Limitations

This study produced evidence of patient–pharmacist interactions, the role of pharmacists and the importance of community pharmacists’ interventions. Evidence was also provided on patient autonomy in community pharmacies. This is important, as it provides information to enhance the role of community pharmacists in terms of medication adherence and quality use of medicines for quality health outcome. Some of the observational studies in this systematic review reported the outcomes of patients who chose treatments based on their preferences and the advice of their provider. However, pharmacist intervention is a factor that influenced treatment choices/decisions of patients, which was also a factor for better health outcome. However, this study had some limitations. First, there was substantial heterogeneity across the included studies; this was likely due to the differences in the adjusted variables across studies and the assessment methods utilized, as some of the studies employed an open-ended questionnaire while others used a closed-ended questionnaire. As a result, a meta-analysis was not performed. Second, although the search strategy was comprehensive, only studies published in English were included.

## 6. Implication for Practice

This review indicates that although patients’ decisions/choices have an impact on the health outcome in terms of medication adherence and quality use of medicines, community pharmacists’ intervention influenced or facilitated the health outcome of patients. Hence, community pharmacists should continue to interact and provide interventions by applying their knowledge, experience, and skills to the best of their ability to assure optimal outcomes for their patients.

## 7. Conclusions

One of the key roles of community pharmacists is to enhance the autonomy of patients to decide/choose their medication management and therapies. Patient–pharmacist interactions contributed to the shared decision-making related to medication management. Patients considered community pharmacists as important health care providers as they provide interventions and help to decide medication adherence and quality use of medicine. The community pharmacists influenced the decision/choice of patients in self-care and self-medication.

## Figures and Tables

**Table 1:** Risk of bias of included study categories is based on Effective Practice and Organization of Care (EPOC).

Study Author, (Year)	Was the Allocation Sequence Adequately Generated?	Was Allocation Adequately Concealed?	Was the Study Adequately Protected Against Contamination?	Was Knowledge of the Allocated Interventions Adequately Prevented?	Were Incomplete Outcome Data Adequately Addressed?	Was Baseline Outcome Measurement Similar?	Was the Baseline Characteristic Similar?	Was the Study Free from Selective Outcome Reporting?	Overall Risk
1. Rickles (2005) [19]	Low	Low	Unclear	Unclear	Low	Low	Low	Low	Unclear
2. Vella (2015) [20]	Unclear	Unclear	Low	Unclear	Low	Low	Unclear	Low	Unclear
3. Robinson (2010) [21]	Low	Low	Low	Low	Low	Low	Low	Low	Unclear
4. Major (2010) [22]	Low	Low	Unclear	Unclear	Low	Unclear	Low	Low	Unclear
5. El-Kohly (2017) [23]	Low	Unclear	Low	Unclear	Low	Low	Unclear	Low	Unclear
6. Kayyali (2018) [24]	Low	Low	Low	High	Low	Low	Low	Low	High
7. Al-Arifi (2012) [25]	Low	Low	Low	Low	Low	Low	Low	Low	Unclear
8. Sookaneknun (2004) [26]	Low	Low	Low	Low	Low	Unclear	Unclear	Low	Unclear
9. Eussen (2010) [27]	Unclear	Low	Unclear	Unclear	Low	Unclear	Low	Low	Unclear
10. Millar (2016) [28]	Low	Low	Low	High	Low	Low	Unclear	Low	Unclear
11. Tinelli (2007) [29]	Low	Low	Low	Low	Low	Low	Unclear	Low	Unclear
12. Gultekin (2019) [30]	Low	Low	Low	High	Low	Low	Low	Low	High
13. Basheti (2008) [31]	Low	Unclear	Low	Unclear	Low	Unclear	Low	Low	Unclear
14. Kelly (2014) [32]	Unclear	Low	Low	Low	Low	Unclear	Low	Low	Unclear
15. Caltic (2013) [33]	Low	Low	Unclear	Low	Low	Low	Unclear	Low	Unclear
16. Merks (2014) [34]	Low	Low	Low	Low	Low	Unclear	Low	Low	Unclear
17. Awad (2017) [35]	Low	Low	Low	Unclear	Unclear	Low	Low	Low	Unclear
18. Wirth (2010) [36]	Low	Low	Low	Low	Low	Low	Low	Low	Unclear
19. Lau (2019) [37]	Low	Low	Low	Low	Low	Low	Low	Low	Unclear

**Table 2:** Characteristics of the included studies.

z	Author	Setting (Country)	Publication Year	Sample Size	Targeted Care Type	Intervention	Outcome Measures	Outcome Assessment
1.	Rickles [19]	USA	2005	60	Pharmacist & Patient	Pharmacist–Patient Collaboration	Patient adherence	Randomized controlled
2.	Vella [20]	Malta	2015	824	Pharmacist & Patient	Pharmacist-led services	Consumer perception	Cross sectional
3.	Robinson [21]	USA	2010	376	Patient	Pharmaceutical care intervention	Medication adherence	Comparison
4.	Major [22]	Hungary	2010	1486	Patient	Habits & Interests	Nonprescription Medications	Cross sectional
5.	El-Kohly [23]	Saudi Arabia	2017	1000	Patient	Patients’ Perception	Perception of Community Pharmacists & Services	Cross sectional
6.	Kayyali [24]	UK	2018	319	Patients	Improving adherence	Shared decision making	Mixed method
7.	Al-Arifi 25]	Saudi Arabia	2012	125	Community Pharmacist	Patient counselling	Pharmacist’s role	Cross sectional
8.	Sookaneknun [26]	Thailand	2004	235	Pharmacist & Patient	Pharmaceutical care intervention	Health outcome	Randomized controlled
9.	Eussen [27]	USA	2010	899	Patient	Community pharmacy-based interventions	Quality use of medicines	Randomized controlled
10.	Millar [28]	UK	2016	539	Community Pharmacists	Intermediate care and medicines management	Pharmacists’ perceptions	Cross sectional
11.	Tinelli [29]	UK	2007	1232	Pharmacists	Medications management service	Pharmacist intervention	Randomized controlled
12.	Gultekin [30]	North Cyprus	2019	110	Patients	Assessment of the source of information	Satisfaction, Techniques and Perception of Information	Cross sectional
13.	Basheti [31]	Australia	2008	97	Pharmacists & Patients	Pharmacist intervention	Quality use of medicines	Randomized controlled
14.	Kelly [32]	Canada	2014	384	Patient	Patient Attitudes	Role & Services of Pharmacists	Cross sectional
15.	Catic [33]	Bosnia-Herzegovina	2013	182	Patient	Patients Perception	Perception and Role of Community Pharmacists	Cross sectional
16.	Merks [34]	UK	2014	417	Patient	Patients’ choice of Pharmacy	Factors that influence patients’ choice	Cross sectional
17.	Awad [35]	Kuwait	2017	433	Patient	Patients Perception, Expectation and Views	Community Pharmacy Practice	Cross sectional
18.	Wirth [36]	Malta	2010	500	Patient	Patients Perception	Perception of the Pharmacists & Services	Cross sectional
19.	Lau [37]	Australia	2019	95	Patient-Pharmacists	Counselling Interactions	Counselling on Persistent Pain	Cross sectional

## Data Availability

The data presented in this study are available on request from the corresponding author. The data are not publicly available due to the nature of the study (i.e., systematic review).
